# Supplementary material for: Using Time Dependent Rate Analysis to Evaluate the Quality of Machine Learned Reaction Coordinates for Biasing and Computing Kinetics
Source: J Phys Chem B. 2025 Oct 8;129(42):10967–74. doi: 10.1021/acs.jpcb.5c04626 (PMC12557361; doi:10.1021/acs.jpcb.5c04626)
Supplement: Supplementary file 1 [file jp5c04626_si_001.pdf]

Supporting information for: Using Time Dependent Rate Analysis to Evaluate the Quality of Machine Learned Reaction Coordinates for Biasing and Computing Kinetics

Nicodemo Mazzaferro*,[1] Suemin Lee*,[2,3] Pilar Cossio,[4,5,a)] Pratyush Tiwary,[2,6,3,b)] and Glen M Hocky[1,7,c)]
[1)] *Department of Chemistry, New York University, New York, NY, 10003, USA*
[2)] *Biophysics Program and Institute for Physical Science and Technology, University of Maryland, College Park, MD, 20742, USA*
[3)] *University of Maryland Institute for Health Computing, Bethesda, MD, 20852, USA*
[4)] *Center for Computational Mathematics, Flatiron Institute, New York, NY, 10010, USA*
[5)] *Center for Computational Biology, Flatiron Institute, New York, NY, 10010, USA*
[6)] *Department of Chemistry and Biochemistry and Institute for Physical Science and Technology, University of Maryland, College Park, MD, 20742, USA*
[7)] *Simons Center for Computational Physical Chemistry, New York University, New York, NY, 10003, USA*

Unbiased rates used in calculating γ

System	Transition Time (s)
FKBP-DMSO	13×10^{-9}
FKBP-DSS	50×10^{-9}
T4 Lysozyme L99A-Benzene	1.3×10^{-3}
Abl Kinase WT-Imatinib	1200
Abl Kinase N368S-Imatinib	400
Abl Kinase L364I-Imatinib	700

TABLE S1: The fixed values of the experimental residence times used in EATR to determine γ during the SPIB iterations. Values taken from Ref. 36.

Fitting k_0 in EATR

We can perform a global fit for the unbiased rate k_0 in addition to γ by calculating the sum of square errors for each (k_0, γ) pair, then taking the minimum. This gives the results in Fig. S1.

Derivation of a relation between γ and $\overline{\tau}_{\text{accel}}$

Considering the observed relationship between the average accelerated time and γ, we provide an argument for why this relationship should exist. We first connect the average accelerated time $\overline{\tau}_{\text{accel}}$ to the average biased simulation time $\overline{\tau}'$:

$$\overline{\tau}_{\text{accel}} = \overline{\tau'\alpha} = \overline{\tau}'\overline{\alpha} + \text{cov}(\tau', \alpha) \ ,$$

$$\overline{\alpha}\,\overline{\tau}' = \overline{\tau}_{\text{accel}} - \text{cov}(\tau', \alpha) \ , \tag{1}$$

[a)] Electronic mail: pcossio@flatironinstitute.org
[b)] Electronic mail: ptiwary@umd.edu
[c)] Electronic mail: hockyg@nyu.edu

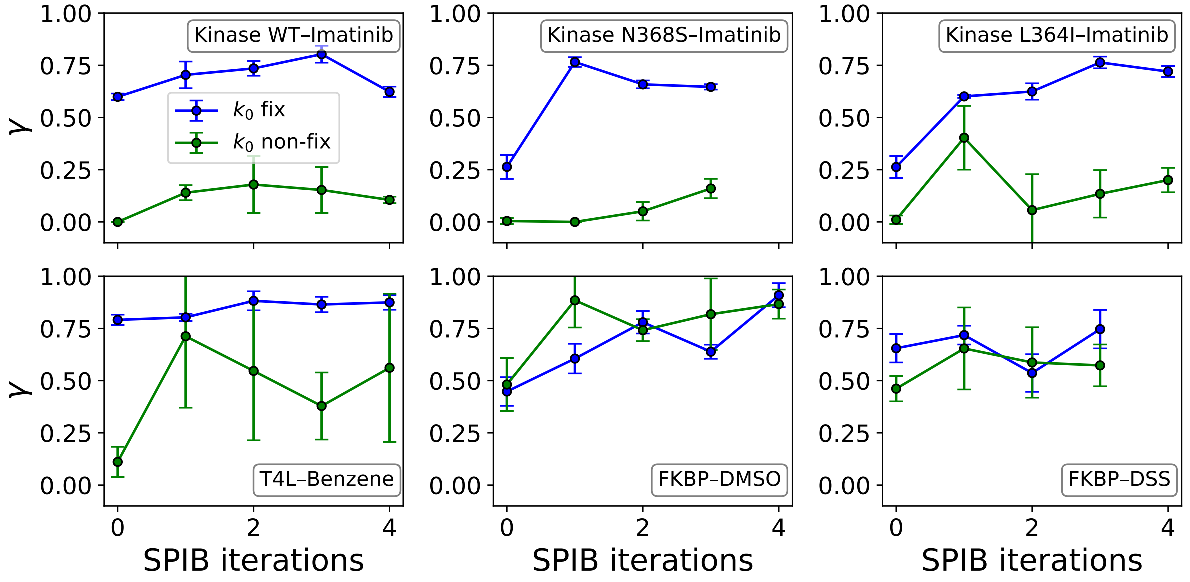

FIG. S1: Comparison of fitted γ values with fixed k_0 versus free k_0. Each subplot shows γ plotted against SPIB iteration for six different protein–ligand systems. Error bars denote the standard deviation of the fitting uncertainty in γ. Blue plot represents γ value in which k_0 was held fixed at its experimental value (as in Fig. 2), while green plot represents γ value in which both k_0 and γ were treated as free parameters.

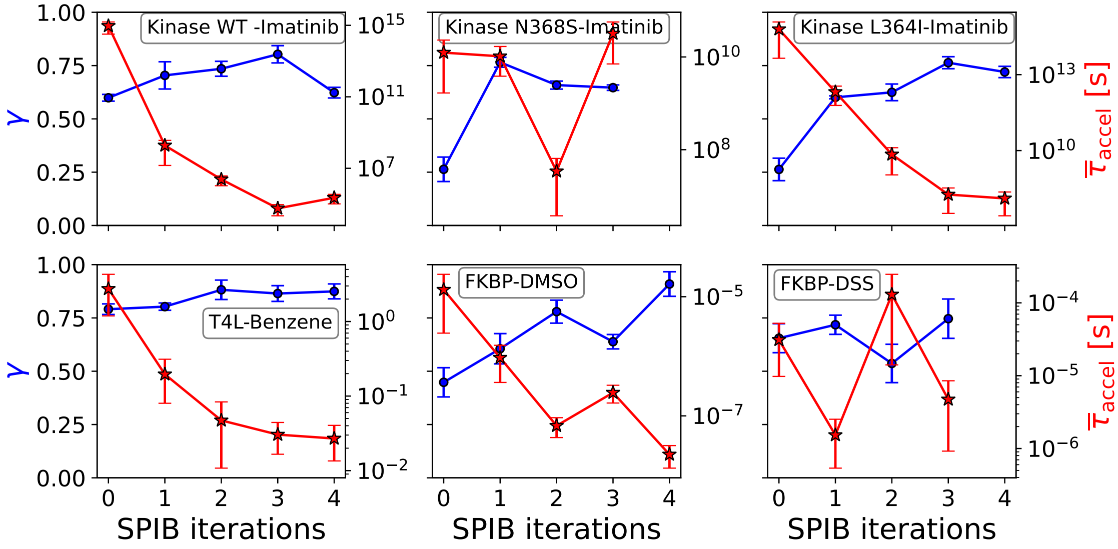

FIG. S2: Same data as Fig. 3 represented in a different way. Here, results of the EATR metric γ (blue circle, left axis) and the mean accelerated time $\overline{\tau}_{\text{accel}}$ (red star, right axis, log scale) over SPIB iterations for six protein–ligand systems with k_0 fixed to the experimental value. Top left to bottom right: ABL kinase Wild Type (WT) - Imatinib, ABL kinase mutant N368S - Imatinib, ABL kinase mutant L364I - Imatinib, T4 Lysozyme - Benzene, FKBP - DMSO, FKBP - DSS. Error bars denote standard deviations of fitting uncertainty in γ and standard deviations in $\overline{\tau}_{\text{accel}}$.

where $\text{cov}(X, Y)$ is the covariance between X and Y.

We now wish to demonstrate a connection between $\overline{\tau}_{\text{accel}}$ and γ. Using the relationship between the biased rate and the biased simulation time, $k = 1/\overline{\tau}'$, and applying Eq. 6, we can rewrite the left hand side of Eq. 1:

$$\overline{\alpha}\,\overline{\tau}' = \overline{\alpha}/(k_0 \overline{e^{\beta\gamma V}}) \qquad (2)$$

To solve for γ, we note that we can approximately take γ outside of the ensemble average for large values of $e^{\beta V}$

(when $0 < \gamma < 1$):

$$\frac{k_0 \overline{e^{\beta\gamma V}}}{\overline{\alpha}} \approx \frac{k_0 \overline{\alpha}^{\gamma}}{\overline{\alpha}} = k_0 \ \overline{\alpha}^{\ \gamma-1}, \tag{3}$$

because $\frac{\overline{e^{\beta\gamma V}} - \overline{e^{\beta V}}^{\gamma}}{\overline{e^{\beta V}}} \approx 0$.

Combining Eqs. 2 and 3 we get,

$$\Rightarrow \overline{\alpha}\,\overline{\tau}' \approx \overline{\alpha}^{\ 1-\gamma} k_0^{-1}. \tag{4}$$

Equating Eqs. 1 and 4 gives us

$$k_0 \left(\overline{\tau}_{\mathrm{accel}} - \mathrm{cov}(\tau', \alpha)\right) \approx \overline{\alpha}^{\ 1-\gamma}$$

and taking the logarithm on both sides and solving for γ, we obtain the following two equations presented in Sec. III B:

$$\gamma \approx 1 - \frac{\log\bigl(k_0(\overline{\tau}_{\mathrm{accel}} - \mathrm{cov}(\tau', \alpha))\bigr)}{\log(\overline{\alpha})} \tag{5a}$$

$$\gamma \approx 1 - \frac{\log\bigl(k_0\, \overline{\tau}_{\mathrm{accel}}\bigr)}{\log(\overline{\alpha})}. \tag{5b}$$
